# Passivation mechanism of thermal atomic layer-deposited Al_2_O_3_ films on silicon at different annealing temperatures

**DOI:** 10.1186/1556-276X-8-114

**Published:** 2013-03-02

**Authors:** Yan Zhao, Chunlan Zhou, Xiang Zhang, Peng Zhang, Yanan Dou, Wenjing Wang, Xingzhong Cao, Baoyi Wang, Yehua Tang, Su Zhou

**Affiliations:** 1 Key Laboratory of Solar Thermal Energy and Photovoltaic System, Institute of Electrical Engineering, Chinese Academy of Sciences, Beijing 100190, China; 2 Institute of Microelectronics, Chinese Academy of Sciences, Beijing, 100029, China; 3 Key Laboratory of Nuclear Analysis Techniques, Institute of High Energy Physics, Chinese Academy of Sciences, Beijing, 100049, China; 4 Shanghai Institute of Technical Physics, Chinese Academy of Sciences, Shanghai, 200083, China

**Keywords:** Thermal ALD, Al_2_O_3_ film, Passivation, Annealing.

## Abstract

Thermal atomic layer-deposited (ALD) aluminum oxide (Al_2_O_3_) acquires high negative fixed charge density (*Q*_f_) and sufficiently low interface trap density after annealing, which enables excellent surface passivation for crystalline silicon. *Q*_f_ can be controlled by varying the annealing temperatures. In this study, the effect of the annealing temperature of thermal ALD Al_2_O_3_ films on p-type Czochralski silicon wafers was investigated. Corona charging measurements revealed that the *Q*_f_ obtained at 300°C did not significantly affect passivation. The interface-trapping density markedly increased at high annealing temperature (>600°C) and degraded the surface passivation even at a high *Q*_f_. Negatively charged or neutral vacancies were found in the samples annealed at 300°C, 500°C, and 750°C using positron annihilation techniques. The Al defect density in the bulk film and the vacancy density near the SiO_*x*_/Si interface region decreased with increased temperature. Measurement results of *Q*_f_ proved that the Al vacancy of the bulk film may not be related to *Q*_f_. The defect density in the SiO_*x*_ region affected the chemical passivation, but other factors may dominantly influence chemical passivation at 750°C.

## Background

Excellent surface passivation is required to realize the next-generation industrial silicon solar cells with high efficiencies (>20%). Silicon oxide films thermally grown at very high temperatures (>900°C) are generally used to suppress the surface recombination velocities (SRVs) to as low as 10 cm/s and applied in front- and rear-passivated solar cells. In recent years, atomic layer-deposited (ALD) aluminum oxide (Al_2_O_3_) thin films have been investigated as candidate surface passivation materials [1-3]. ALD Al_2_O_3_ thin films enable perfect passivation similar to high-quality thermally grown silicon oxide and can be prepared at low temperatures (<300°C). Given that the silicon bulk lifetime is sensitive to high temperatures, ALD Al_2_O_3_ has a natural advantage over thermal SiO_2_ in terms of integration into industrial cell processes. Extensive experiments on Al_2_O_3_ film applications in photovoltaics have demonstrated that Al_2_O_3_ can passivate both low-doped n- and p-type silicons. ALD Al_2_O_3_ also exerts a better passivation effect on p^+^-type emitters than other dielectric layers. Very recently, Hoex et al. [4] found that Al_2_O_3_ can also enable high-surface passivation for n^+^-type emitters within the range of 10 to 100 Ω/sq.

Low SRVs for dielectric passivation are attributed to two passivation mechanisms: chemical passivation and field-effect passivation [5,6]. Chemical passivation (e.g., thermal SiO_2_ films) decreases the interface defect density (*D*_it_). In dielectric layers such as SiN_*x*_ and Al_2_O_3_, a high fixed charge density (*Q*_f_) near the silicon surface generates an electric field, repelling electrons or holes to reduce carrier recombination on the surface. Thermal ALD Al_2_O_3_ reportedly acquires a negative *Q*_f_ as high as 10^13^ cm^-2^ with sufficiently low *D*_it_ (about 10^11^ eV^-1^ cm^-2^) after annealing [7,8]. Experiments have shown that the fixed charge located near the Al_2_O_3_/Si interface is related to some types of defect proposed as Al vacancies, interstitial O, and interstitial H in Al_2_O_3_ film or at the interface [5]. Positron annihilation is a useful technique for vacancy-type defect investigation. Edwardson et al. [9] performed Doppler broadening of annihilation radiation (DBAR) studies and found an interface that traps positrons in an ALD Al_2_O_3_ sample, which significantly differed from the *S*-*W* result of DBAR in the current work. The discrepancy can be attributed to the different annealing conditions.

In the present study, the effect of annealing temperature on the surface passivation characteristics of Al_2_O_3_ films was investigated. Corona charging experiments were performed to distinguish between chemical and field-effect passivation mechanisms. Slow positron beam DBAR measurements were performed to probe the defects in Al_2_O_3_ films annealed at 300°C, 500°C, and 750°C.

## Methods

### Experimental

Aluminum oxide films were deposited onto a 1 to 10 Ωcm p-type Czochralski Si (100) substrate using the thermal ALD method. The 420-μm-thick double-sided polished wafers were cleaned using the RCA standard method and dipped in 1% hydrofluoric acid for 1 min before deposition to remove the native oxide layer on the surface. Thermal ALD Al_2_O_3_ films about 23 nm thick were prepared with Al(CH_3_)_3_ and H_2_O as reactants at 250°C. The optimum deposition temperature that led to the highest as-deposited effective lifetime was determined to be 250°C. Double faces were deposited to prepare symmetrical Al_2_O_3_/Si/Al_2_O_3_. After deposition, the samples were annealed at different temperatures (300°C to 750°C) for 10 min in air. Annealing in air was performed because it closely resembles the firing condition in the manufacturing process of solar cells. The effective lifetimes of these samples were measured before and after annealing, and a negative *Q*_f_ of the Al_2_O_3_ films was obtained using corona charging measurements using Semilab WT2000 (Semilab Semiconductor Physics Laboratory Co. Ltd., Budapest, Hungary). DBAR measurements of the three annealed samples (300°C, 500°C, and 750°C) were performed to investigate the defects in the films. A slow beam of positrons that had variable energies (<10 keV) was used to obtain information from the thin films.

### Corona charging measurement

**Figure 1:**
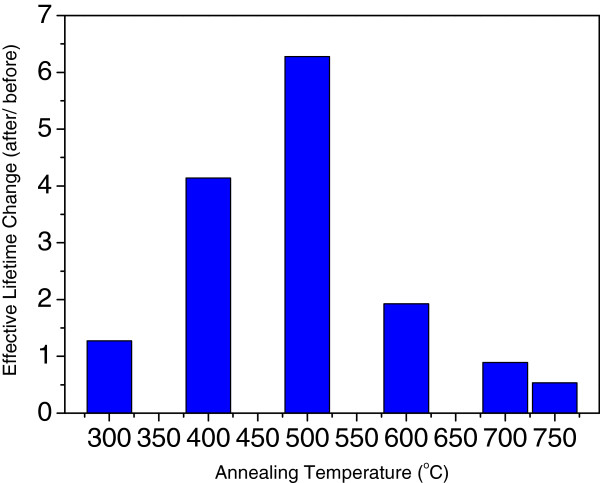
**Typical corona charging measurement for the as-deposited Al**_**2**_**O**_**3**_**/Si sample.**

The effective lifetime of the annealed samples was measured using the microwave photoconductive decay method. Corona charging experiments were performed to determine *Q*_f_[10]. As a positive charge was added stepwise to the film surface using a corona, the effective lifetime decreased until the positive charge was totally balanced with the negative fixed charge and then increased because the positive charge also enabled field-effect passivation. Thus, the negative *Q*_f_ was equal to the amount of added corona charge density (*Q*_c_) at the minimum point of the *τ*_eff_-*Q*_c_ curve. The surface passivation mechanism comprises chemical passivation and field-effect passivation. Thus, the minimum effective lifetime was also obtained to determine the role of chemical passivation because the effective lifetime is mainly controlled by chemical passivation when the negative charge is neutralized. Figure 1 shows the typical corona charging measurement for the as-deposited Al_2_O_3_/Si sample. *Q*_f_ before annealing was determined as -3.5 × 10^11^ cm^-2^ from the curve, and the lowest lifetime was recorded as 42.8 μs to characterize the chemical passivation of the sample.

### DBAR measurement

Positron annihilation is used to analyze defects in oxides and semiconductors [11-13]. When a positron is implanted into a matter, it annihilates an electron and emits two γ rays. The energy of γ rays varies around 511 keV because of the energy and momentum conservation of the positron-electron system given by the relation *E*_γ_ = 511 ± Δ*E*_γ_ keV, where Δ*E*_γ_ is the Doppler shift. Even a slight change in momentum can lead to a large shift of energy. The *S* and *W* parameters were calculated to characterize Doppler broadening. The *S* parameter is defined as the ratio of the mid-portion area to the entire spectrum area. The *W* parameter is the ratio of the wing portion to the entire area. With increased concentration of vacancy in solid, the positron is mostly trapped and annihilates low-momentum electrons, leading to a narrow Doppler peak with a high *S* parameter. *W* parameters are higher and *S* parameters are lower when annihilation of the core electrons of atoms occurs. Given that the momentum distribution of electrons varies in different types of defect, changes in *S*-*W* plots can also characterize the types and distributions of defects in the films [14].

## Results and discussion

### Influence of annealing temperature on surface passivation

**Figure 2:**
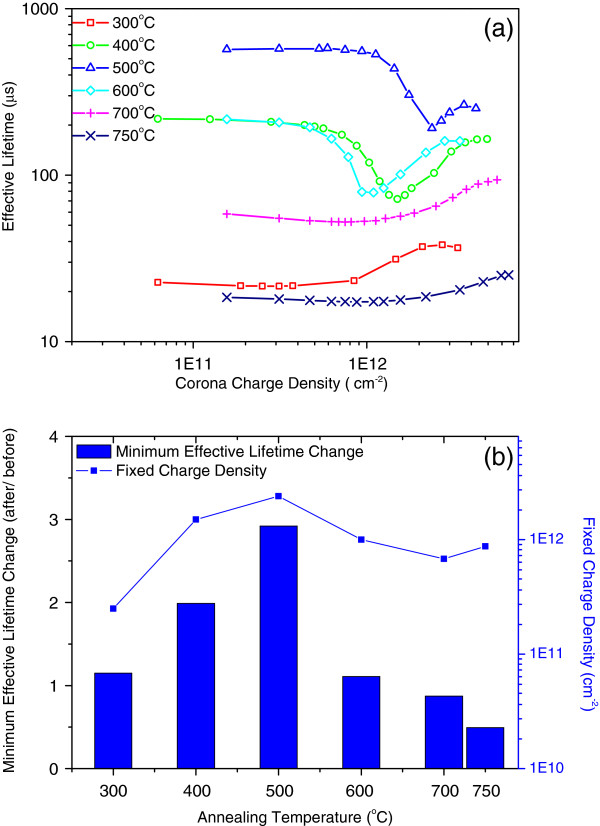
**Influence of annealing temperature on Al**_**2**_**O**_**3 **_**passivation.**

The effective lifetimes of the samples annealed at different temperatures in air are shown in Figure 2. The effective lifetime change is the ratio of the effective lifetime after annealing to that of the effective lifetime before annealing. The ratio was used instead of the actual value because the effective lifetimes of the six as-deposited samples (before annealing) were not strictly identical, which rendered meaningless the observation of the absolute value of the effective lifetime after annealing. The effective lifetime change initially increased with increased annealing temperature and then rapidly decreased below unity. This result indicated that passivation collapsed at annealing temperatures higher than 700°C. The optimum annealing temperature was around 500°C in air, which was higher than the reported 400°C to 450°C when annealed in N_2_[15].

**Figure 3:**
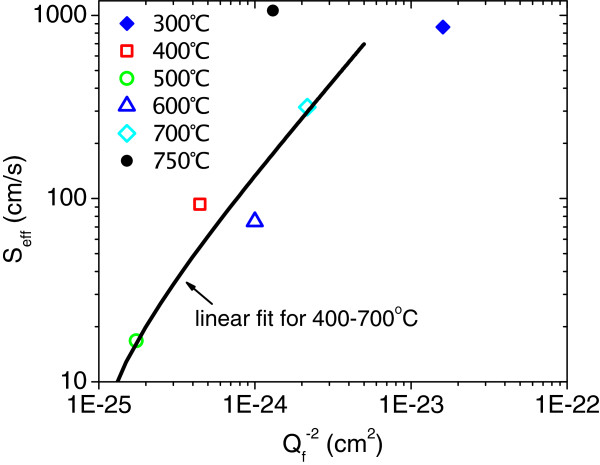
**Corona charging measurement of samples. (a) **Before and after annealing. **(b) **Fixed charge density and minimum effective lifetime change after annealing at different temperatures.

Corona charging measurement was performed to observe the field-effect and chemical passivation mechanisms. *Q*_f_ and the lowest lifetime can be extracted from the resulting measurement curve, as described in the section ‘Corona charging measurement.’ Figure 3a shows the measured data, and Figure 3b shows the *Q*_f_ and the minimum effective lifetime change (lowest lifetime after annealing vs. as-deposited value) as a function of the annealing temperature. *Q*_f_ significantly increased to 10^12^ cm^-2^ after annealing at 400°C compared with *Q*_f_ of about 10^11^ cm^-2^ before annealing (Figure 1). *Q*_f_ increases from 2.5 × 10^11^ cm^-2^ at 300°C, reaches the highest point of about 2.5 × 10^12^ cm^-2^ at 500°C, and thereafter decreases to 8 × 10^11^ cm^-2^. *Q*_f_ did not significantly change when the annealing temperature was higher than 600°C. Meanwhile, the effective lifetime of the sample annealed at 300°C was slightly enhanced (Figure 2), i.e., 1.2 times greater than that of the as-deposited sample. This result indicated that *Q*_f_ of 2.5 × 10^11^ cm^-2^ did not significantly affect surface passivation. The chemical passivation variation at 300°C to 500°C was similar to *Q*_f_ based on the minimum lifetime in the corona charging measurement. The chemical passivation effect increased with increased annealing temperature before 500°C and quickly decreased thereafter. This variation was related to the hydrogen release from the film found by Dingemans [16].

Notably, *Q*_f_ reached 10^12^ cm^-2^ after annealing at 750°C, and this value was almost one magnitude higher than that of the as-deposited sample. However, the effective lifetime was low (Figure 2) because of the poor chemical passivation at 750°C in Figure 3b of the minimum lifetime change value. Therefore, chemical passivation was a prerequisite in achieving excellent surface passivation.

**Figure 4:**
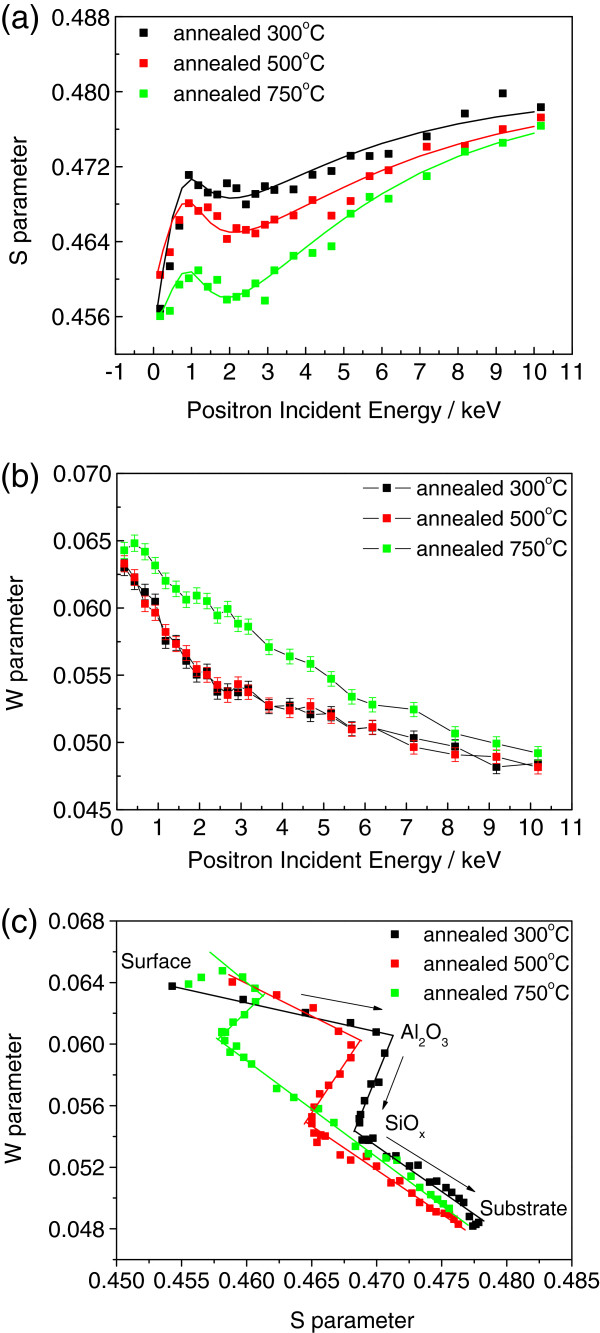
**Plot of *S***_**eff **_**and 1/*Q***_**f**_^**2 **^**with the linear fit for annealing temperatures. **The annealing temperatures are between 400°C to 700°C (*Q*_f_> 6.8 × 10^11^cm^-2^). The slightly bent linear fit line was due to the logarithmic *X*- and *Y*-axes.

The approximate effective lifetime *τ*_eff_ of a symmetrically passivated silicon wafer can be expressed as 1/*τ*_eff_ = 1/*τ*_b_ + 2*S*_eff_/*W*, where *τ*_b_ is the bulk lifetime, *W* is the crystalline silicon (c-Si) wafer thickness, and *S*_eff_ is the effective SRV. The bulk lifetime was estimated at about 1 ms using the I_2_ passivation method to determine *S*_eff_. Figure 4 shows that *S*_eff_ was linear with 1/*Q*_f_^2^ for negative *Q*_f_ values >6.8 × 10^11^ cm^-2^, except for the sample annealed at 750°C. The linear relationship of samples annealed between 400°C and 700°C indicated that passivation was dominated by field-effect passivation (*Q*_f_). Thus, the sample annealed at 300°C (dislocated line) indicated that *Q*_f_ of 2.5 × 10^11^ cm^-2^ was too low to dominate surface passivation, which confirmed the conclusion drawn from Figure 3. This result also agreed with the simulation of Hoex et al. for p-type c-Si [5]. Based on the dislocation of the sample annealed at 750°C, a high interface trap density was inferred to destroy the field-effect passivation and increase *S*_eff_.

### DBAR analysis at different annealing temperatures

DBAR analysis was performed at the Beijing Slow Positron Beam (Institute of High Energy Physics, Chinese Academy of Sciences, Beijing, China). A positron beam generated from a Na^22^ radioactive source was used, and the energy of the positrons was modulated between 0 and 10 keV to obtain the incident energy profile of positron annihilation. The energy region of the *S* parameter ranged from 510.24 to 511.76 keV, whereas the *W* parameter ranged from 504.2 to 508.4 and from 513.6 to 517.8 keV. Thus, the total energy region of the peak ranged from 504.2 to 517.8 keV.

**Figure 5:**
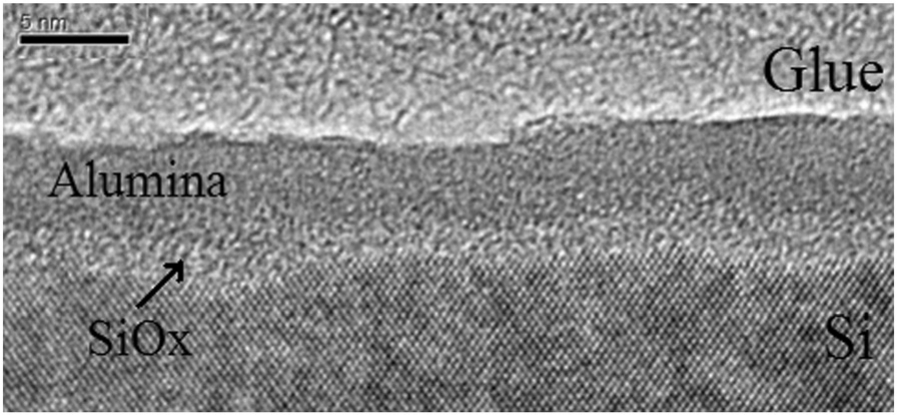
**Doppler broadening spectroscopy of *S*-*W *parameters vs. positron incident energy. (a)***S *and **(b)***W *parameters vs. positron incident energy for samples annealed at different temperatures for 10 min. **(c)***S*-*W* plot for samples annealed at different temperatures for 10 min.

The vacancy defects in the alumina films were mainly Al vacancies, O vacancies, and clusters of vacancies (voids) [13,17,18]. O vacancies with a positive charge (F^+^- and F^2+^-type defects) have difficulty trapping positrons because of their identical charge. Nobuaki Takahashi et al. [19] calculated the defect energetics using first-principle calculations and found that the oxygen vacancy has a much higher formation energy than the aluminum vacancy [19], further supporting the view that few positrons are trapped in charged O vacancies. Therefore, Al and neutral O vacancies (F center) are crucial to the annihilation results in the present study. Figure 5a,b shows the measured *S* and *W* parameters as a function of the incident positron energy for samples annealed at different temperatures for 10 min. In Figure 5a, the shapes of the three curves are similar because the deposition conditions of the three films were identical, and the substrates on which these films grew were also the same. The first one or two points were recorded at low positron incident energy (<0.5 eV), which can be ascribed to the trap states near the film surface. The *S* parameter of the injection energy was approximately between 0.5 and 2 keV, which mainly represented the annihilation events occurring in the aluminum oxide film. Figure 5a shows that the *S* parameter initially increased rapidly, which indicated a higher vacancy defect density of the inner oxide film than that of the surface. A decrease was observed beyond 1 keV, demonstrating that the *S* parameter of the Al_2_O_3_/Si interface was lower than that of the Al_2_O_3_ films. The lower *S* parameter can be attributed to the positron annihilation with high-momentum electrons of oxygen at the interface. This result was probably due to the SiO_*x*_ layer grown between the aluminum oxide and Si substrate, which reportedly has an important function in excellent surface passivation [6,20,21]. The *S* parameter continued to increase after 2 keV with increased incident energy because larger portions of positrons were injected into the silicon substrate. The *S* parameter in the substrate was much higher than that in the oxide film because of the different chemical environments of annihilation. The *S* parameter did not reach a constant value before 10 keV, which implied that positrons with 10 keV energy cannot completely penetrate the Si substrate far from the oxide layer. The *S*-*E* plot in Figure 5a also shows that the *S* parameter in Al_2_O_3_ films (about 1 keV) evidently decreased with increased annealing temperature because of the decreased density of trap vacancies in the Al_2_O_3_ films. The *W* parameter was more sensitive to the chemical environment of the annihilation site. The larger *W* and smaller *S* parameters indicated more positrons annihilating core electrons. Thus, the smallest *S* and largest *W* parameters of the sample annealed at 750°C (Figure 5a,b) implied that the Al_2_O_3_ films had been compressed at this temperature with the lowest vacancy defect density and that the film structure probably did not change.

The *S* and *W* parameters of the same incident energy were plotted in one graph, as shown in Figure 5c. The *S* vs. *W* diagrams of monolithic materials present clusters of points because all *S* or *W* parameters are almost the same [14]. For example, in one type of defect, the *S* and *W* parameters may vary with the positron incident energy, and the *S*-*W* plot extends to the line passing the data point of the bulk region without defect [13,14]. The slope of the line changes with the layers of different compositions and defect types. Thus, the annealed sample consisted of a three-layered structure in which each curve consisted of three extended line segments (Figure 5c). This finding corresponded with the *S*-*E* curve analysis result, which also suggested that the film contained a layer different from Al_2_O_3_ and Si near the interface. No significant interface response in the *S*-*W* result has been previously observed [9], and the discrepancy may be a result of the different annealing environments (air vs. N_2_). Annealing in air may lead to a thicker interface oxide (SiO_*x*_) resulting in more evident responses in the DBRA result.

The different slopes of the Al_2_O_3_ segment of the three samples indicated that the defect types or chemical environments of these samples were different. The three lines crossed one another to avoid passing through a single point of bulk sample without defects, indicating that each of the samples had more than two types of defect. As mentioned in the section ‘DBAR analysis at different annealing temperatures,’ the *S* parameter was mainly influenced by Al and neutral O vacancies. Thus, residual C during deposition and O-H bond content also possibly influenced the *S*-*W* line slope. Residual C varied with the annealing temperature and may have thus influenced the environment of Al vacancies, although further investigations are needed.

**Figure 6:**
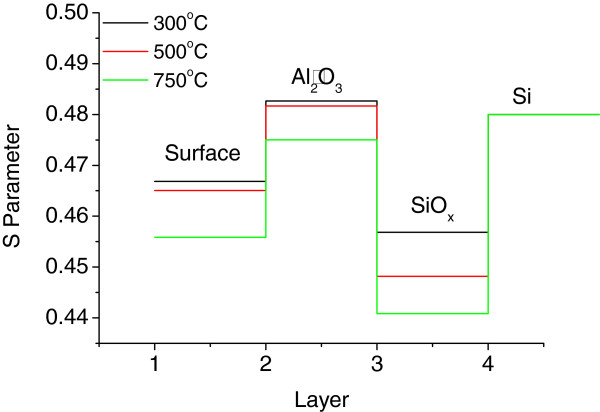
TEM image of aluminum oxide films prepared using thermal ALD.

A thinner sample was prepared to understand the microstructure of the Al_2_O_3_/Si samples, which showed a three-layered structure in DBAR analysis. The 6-nm-thick sample was obtained using thermal ALD and observed by transmission electron microscopy (TEM). The result in Figure 6 shows three layers, namely Si, Al_2_O_3_, and Si-Al_2_O_3_ interface layers, which have been reported for nonstoichiometric silica (SiO_*x*_) [6,20,21].

**Figure 7:**
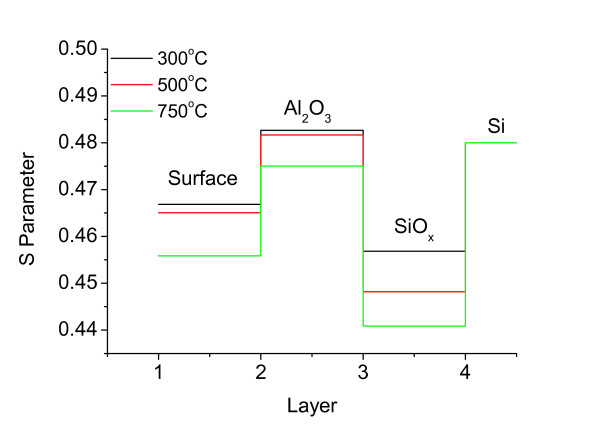
**Fitted *S *parameters for different positions in Al**_**2**_**O**_**3 **_**films using the VEPFIT program.**

The fitted *S* parameter can be clearly analyzed in different parts of a film to gain accurate information from DBAR spectroscopy. In this study, the energy of injected positrons had a different distribution at the positron incident energy of the *X*-axis in the *S*-*E* plot. The positrons also reached different layers of the film. Thus, the *S* parameter of each point in the *S*-*E* plot contained integrated information on multiple layers. The *S* parameter was separated in different layers, and the density/type of vacancies was analyzed at different positions in the film. The *S*-*E* plot was fitted using the VEPFIT program to calculate the *S* parameter from different layers using a four-layered mode, which corresponded to the surface/Al_2_O_3_/SiO_*x*_/Si structure observed by TEM. The obtained *S* parameter is shown in Figure 7. The *S* parameter in the Al_2_O_3_ films decreased with increased temperature, indicating that the vacancy density in the Al_2_O_3_ film decreased with increased annealing temperature. The *S* parameter was much lower in the SiO_*x*_ layer than that in Al_2_O_3_ and the Si substrate. The *S* parameter also decreased with increased annealing temperature, which probably corresponded with the dominant P_b_ defect that decreased with increased annealing temperature [22].

Al vacancies, O interstitials, and H interstitials are proposed as the reasons for the negative *Q*_f_ of Al_2_O_3_[23,24]. The measured *Q*_f_ in Figure 3 and information on Al vacancies in Figure 7 were considered in analyzing the effect of Al vacancy density on the negative fixed charge *Q*_f_. With increased annealing temperature from 300°C to 500°C, the increase in *Q*_f_ was opposite to the decrease in Al vacancy in the bulk film. Thus, *Q*_f_ may not be related with Al vacancies in the Al_2_O_3_ films. The measured minimum effective lifetime in Figure 3 and S parameters of SiO_*x*_ interface in Figure 7 were correlated, and the decrease in vacancy of SiO_*x*_ was coincident with the enhanced chemical passivation at annealing temperatures lower than 500°C. However, the chemical passivation breakdown at 750°C cannot be explained: among the samples annealed at 300°C and 750°C, the chemical passivation at 750°C was the poorest, but the defect density at the interface region still decreased. The functions of interstitial atoms (O or H) near the interface require further investigation.

## Conclusions

*Q*_f_ did not significantly affect the passivation at a low annealing temperature (300°C). The interface trap density markedly increased at a high annealing temperature (750°C) and failed at surface passivation even at a high *Q*_f_. Positron annihilation techniques were used to probe the vacancy-type defects. A three-layered microstructure of thermal ALD Al_2_O_3_ films on Si substrate was found. The Al defect density in the bulk film and the vacancy density near the interface decreased with increased temperature based on the fitted *S* parameter at different positions in the Al_2_O_3_ films. The Al vacancy of the bulk film was not related to *Q*_f_ based on the *Q*_f_ measurement results. The effects of interstitial atoms on *Q*_f_ need further investigation. The defect density in the SiO_*x*_ region may affect chemical passivation, but other factors may also influence chemical passivation particularly beyond 500°C.

## Competing interests

The authors declare that they have no competing interests.

## Authors' contributions

YZ participated in the design of the study, carried out the fabrication of Al2O3 films, performed the statistical analysis, as well as drafted the manuscript. CLZ designed the study to find the relation between negative-charged Al vacancy and *Q*_*f*_. XZ carried out the TEM analysis and participated in the *Q*_*f*_ test. YND performed the film deposition. PZ, XZC, and BYW provided the Beijing Slow Positron Beam and performed the positron BDAR analysis. WJW, YHT, and SZ co-wrote the paper. All authors read and approved the final manuscript.
